# Comparison between Different Types of Sensors Used in the Real Operational Environment Based on Optical Scanning System

**DOI:** 10.3390/s18061684

**Published:** 2018-05-24

**Authors:** Wendy Flores-Fuentes, Jesús Elías Miranda-Vega, Moisés Rivas-López, Oleg Sergiyenko, Julio C. Rodríguez-Quiñonez, Lars Lindner

**Affiliations:** 1 Facultad de Ingeniería, Universidad Autónoma de Baja California, Mexicali, Baja California 21280, Mexico; flores.wendy@uabc.edu.mx (W.F.-F.); julio.rodriguez81@uabc.edu.mx (J.C.R.-Q.); 2 Instituto de Ingeniería, Universidad Autónoma de Baja California, Mexicali, Baja California 21280, Mexico; mrivas@uabc.edu.mx (M.R.-L.); srgnk@uabc.edu.mx (O.S.); lindner.lars@uabc.edu.mx (L.L.)

**Keywords:** light emitting diode (LED-receiver), photodiode, real environment, light dependent resistor (LDR), optical scanning system (OSS)

## Abstract

The present paper describes the experimentation in a controlled environment and a real environment using different photosensors, such as infrared light emitting diode (IRLED-as receiver), photodiode, light dependent resistor (LDR), and blue LED for the purpose of selecting those devices, which can be employed in adverse conditions, such as sunlight or artificial sources. The experiments that are described in this paper confirmed that the blue LED and phototransistor could be used as a photosensor of an Optical Scanning System (OSS), because they were less sensitive to sunlight radiation. Moreover, they are appropriate as reference sources that are selected for the experiment (blue LED flashlight and light bulb). The best experimental results that were obtained contained a digital filter that was applied to the output of the photosensor, which reduced the standard deviation for the best case for the phototransistor LED from 100.26 to 0.15. For the best case, using the blue LED, the standard deviation was reduced from 86.08 to 0.11. Using these types of devices the cost of the Optical Scanning System can be reduced and a considerable increase in resolution and accuracy.

## 1. Introduction

One of the principal targets of an Optical Scanning System (OSS) is the noise reduction under real-life conditions. There are several possible sources of interference, which may cause problems when the system is exposed to adverse environmental conditions. The main sources of noise are sunlight, electrically or magnetically induced interferences, and electronic components, such as Op-amps that are used in the OSS to measure and amplify small signals, as the 60 Hz power line frequency, which is a source of substantial noise in many photosensors. On the other hand, when photodetectors are used to evaluate the performance and the accuracy of an optical system, it is critical to take into a consideration both the sensor and the light source. It is also important to consider that all commercial photosensors are sensitive to sunlight due to their spectral response of the sun. In practice, however, it is possible to find solutions using filters, such as optical filters, to attenuate the undesired wavelength radiation and analog filters to remove the electrical noise. In addition, to solve these kind of problems, there are methods and sophisticated techniques, such as computational statistics and digital filters, which can be used to reduce the noise from environmental sources. These methods and devices can improve the performance of optical scanning systems. It is important to mention that these methodologies are based on the quality of the data. The authors in [1] reviewed the different aspects of SHM in detail, such as feature extraction and statistical analysis.

The OSS is based on optoelectronic sensors to capture an optical signal from a specific point in space and the main reason is because it is necessary to obtain the shape of an object or to monitor this point under observation. Devices, such as LED, phototransistor, photodiode, and light-dependent resistor (LDR) can be used as a natural filter due to their narrow electromagnetic spectrum, and which are available on the commercial market at low cost. However, choosing these types of sensors depend on the application, for example when the OSS is not influenced by other source of radiation. There are two types of techniques for remote object sensing, which are divided into active and passive methods [2]. Laser scanning systems as an active method for object scanning, which employs its own energy source [3]. The important advantage of using Laser Scanning Systems is that this system can control its own source of radiation and it is also possible to obtain measurements anytime.

Passive methods detect natural energy that is reflected or emitted from the observed object. Also, the OSS can work with an incoherent source light, such as light emitting diode (LED) or coherent light, such as laser. The advantage of this method is that the cost of the overall system is cheaper than the active method when compared to the active method. Another important advantage of the passive method is that it can be used to monitor a structural performance by placing on the structure a source of radiation, such as a light bulb, LED, or Laser. Nowadays, the development of optoelectronic sensors that can be used in adverse conditions, such as sunlight and other sources of radiation, represents a challenge, particularly in the industrial applications of structural health monitoring (SHM), navigation of autonomous mobile robots, remote sensing, and any other application that requires sensing the light from an object. The principle of operation of the passive method is that the object being observed reflects ambient radiation that is emitted by the sun or a nearby source of artificial light [4].

Photodiodes and photoresistors, such as light-dependent resistor (LDR), are an example of photodetectors [5]. The use of optoelectronic devices can provide a natural filter, due that they operate within a specific range of wavelength, this is important to compare each device mentioned before in this paper because under real-life environmental conditions some sensors not working well. Photodiode and phototransistors are widely used as optoelectronic sensors for optical scanning. However, in [6,7] the LED has been used as a photosensor with satisfactory results. The photosensor mentioned before is used as receiver of a light that is reflected from the object and can be found most commonly in scanning systems for three-dimensional (3D) vision-based range scanning tasks.

Paper [8] proposed a novel positioning system for indoor application, which can measure the angular position of a moving optical sensor by using visible light communication, photodiodes, and flickering infrared LEDs. Working under real-life environmental conditions, the accuracy of this can be affected by the interference of the sun, because its spectral response due to the infrared used responds to 940 nm. The infrared LED can be changed by far infrared LED, the accuracy and resolution may be better in spite of adversity of the environmental conditions that are caused by the sunlight. In [9], the photodiode has been used for spatial measurements using optical scanning in a controlled environment. There are other examples where there are applied photosensors, such as photodiodes, for example, OPT301 in [10]. However, when it comes to realizing the experiments in a real environment, the measurements are affecting the accuracy and precision of the OSS [11]. When the devices are near to visible light, it is most difficult to discriminate the signals that are generated by the sunlight and external sources. To solve this kind of issue, the photosensor uses a daylight blocking filter in order to select a specific operating wavelength.

The present paper only deals with passive methods, and the principal elements that are used are photosensors, such as photodiodes, blue and infrared LEDs as photosensors, and light dependent resistors (LDR). The main goal of this paper is discriminating the optical noise that is generated in a real environment caused by the sunlight with devices that are widely available and inexpensive. Another challenge that will be considered in this works belongs to the field of the OSS, that is the electrical noise that appears mainly in digital and analog circuits, and because it is important conditioning the signal before to process it. In [12], were applied the digital filters, such as median filter and moving average filter, for the conditioning of the scattered light signals of the three photodiodes that were employed in that work. In addition, transimpedance amplifier can be used to amplify the signal that is sensed by the photodiode.

## 2. Theoretical Fundamentals of the Photosensors

A photosensor converts electromagnetic radiation into a different physical form, usually electrical charge [13], which are then classified into two major categories: photoelectric sensors and thermal sensors [14]. It must be emphasized that the present work is based on photoelectric sensors mainly made of semiconductor materials such as: photodiodes, light emitting diodes (LED) and LDRs. To understand how photoelectric sensors works, it is important to understand the photoelectric effect. According to [15], the observation of the photoelectric effect occurs by incident light on a metal surface, which causes the emission of electrons from this surface. Also, the amount of the emitted electrons and their velocity can be measured. Depending on the color of the light, the electron speed can change, for example, when the color of the light is red, the electron speed is slow when compared with the blue light. Equation (1) shows the relation between the energy gap of material and frequency of the photon.

(1) $$hv\geq E_{g}$$

where h is Planck’s constant, v is the frequency of the photon and $E_{g}$ is the energy gap or band gap, known as energy difference between the valence band and the conduction band.

By knowing $v=\frac{c}{\lambda }$, Equation (1) can be represented in term of wavelength λ.

(2) $$\lambda \leq \;\frac{hc}{E_{g}}.$$

Equation (2) reveals the relationship between the wavelength and the bandgap of the optoelectronic sensor based on semiconductor. For example, the energy gap $E_{g}$ of silicon and germanium is 1.12 eV and 0.66 eV, respectively. Table 1 illustrates the principal parameters of the semiconductors materials that are used as photosensors.

## 3. Optical Scanning System

**Figure 1:**
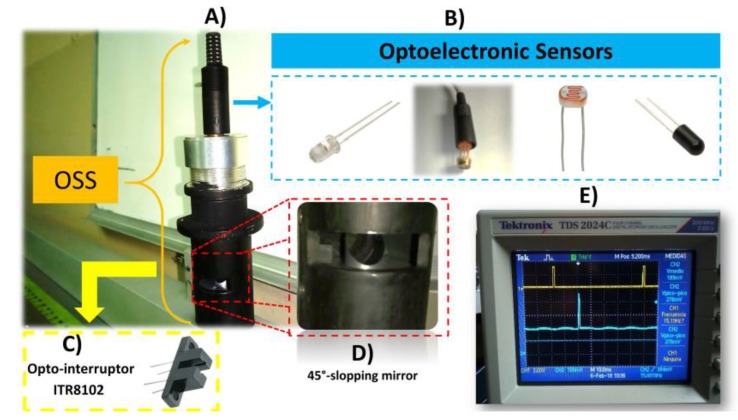
Developed Scanning Aperture, this figure shows the principal elements of the scanning systems. The elements are listed as: (**A**) Scanning Aperture; (**B**) Optoelectronic sensor that used (light emitting diode (LED) photodiode OPT301 and light dependent resistor (LDR)); (**C**) Opto-interruptor ITR8102; (**D**) Slopping mirror; and, (**E**) Oscilloscope displaying the signals generated by pulses of ITR8102 (yellow) and Gaussian signal (turquoise).

In the optoelectronic and measurements laboratory of the Engineering Institute of Universidad Autónoma de Baja California, a method for the task of SHM by using an OSS has been developed. On the other hand, one of the principal targets to increment accuracy and resolution for this system is reducing the noise that is caused by sunlight and environmental conditions, due to those operational conditions that affect measured signals during data acquisition. In the field of SHM, the OSS plays an important role to measure structural deformation and displacements of buildings to monitor and prevent undesirable damage. Additionally, the OSS has several applications such as 3D & 2D machine vision technologies, medical scanning, and its necessary accuracy and resolution in each activity in order to measure minimal changes to assess structural conditions [16]. The developed OSS consists principally of devices and elements, such as a non-rotating incoherent light emitter that is mounted on the structure, a 45°-slopping mirror inside of the OSS, a double convex lens, and an optoelectronic sensor as a photodiode, as shown in Figure 1:

When two scanning apertures are used, the system can calculate coordinates and distances between the source of light and OSS. Each peak of the Gaussian curve is related to the angle of position of the source, in this way, the angle $B_{i}$ for the SA #1 and the angle $C_{i}$ for the second SA #2 are obtained. When considering that the distance a between the apertures is known, the coordinates of the object or structure to analyze can be calculated by using theorems of sines and the correlation between the sides in the triangle.

However, in the present work, the different types of optoelectronics sensor were tested in a controlled environment (laboratory) and a real operational environment, in order to compare the accuracy and resolution of the OSS. The environment conditions affect the measurements of the angles of each SA, the principal source of noise and the loss of resolution from the optical scanning system is the excessive bright of sunlight, as previously mentioned. In [9,17], is presented a complete description of the device system principle of operation. According to these works, the OSS generates a Gaussian signal, where the peak of this signal is related to the center of energy. Once knowing the center of energy, the angle is measured, as follows.

The distance of is *T*_2*π*_ equal to the time between *M*_1_ and *M*_3_, expressed in Equation (3).

(3) $$T_{2\pi }=M_{3}-M_{1}$$

On the other hand, the time $T_{\alpha }$ is equal to the distance between $M_{1}$ and $M_{2}$, as expressed by Equation (4).

(4) $$T_{\alpha }=M_{2}-M_{1}.$$

where *T*_2*π*_ and *T_α_* are expressed in samples. With this consideration, the time variable can be eliminated from Equation (4) and α, thereby is calculated as follows.

(5) $$\alpha =\frac{2\pi \cdot T_{\alpha }}{T_{2\pi }}$$

**Figure 2:**
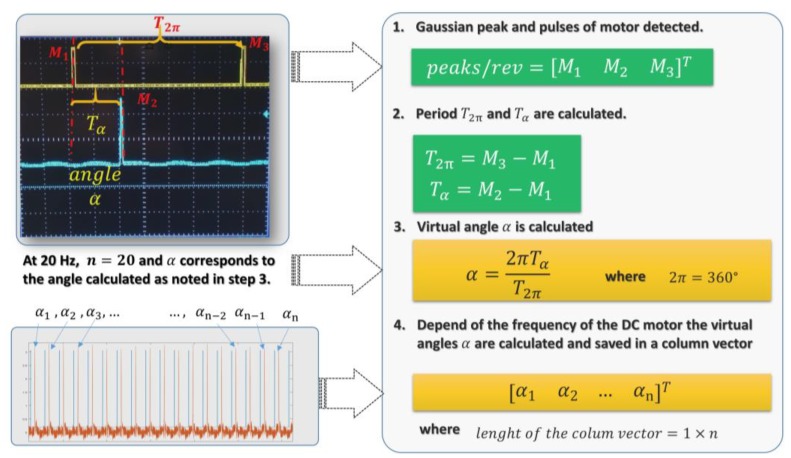
Procedure of measuring of the virtual angle between the photosensor and the reference source (light bulb/LED flashlight).

The procedure for measuring the virtual angle α is shown in Figure 2. For example, in step 1, the number of the rising and falling edge that is generated by the encoder in the variable *M*_1_ and *M*_3_ is recorded. The measurement that is shown in step 2 corresponds to the time of one scanning cycle and the difference between the starting pulses of motor and peak of the Gaussian signal by applying Equations (5) and (6), respectively. Once *T*_2*π*_ and *T_α_* are determined, the virtual angle α can be calculated, as shown in step 3 using Equation (7). Depending on the frequency of the DC motor, the virtual angles are stored in a column vector where the length of the vector is 1 × *n*, and *n* is equal to the scanning frequency, according to step 4. For example, the scanning frequency of the DC motor is 20 Hz, which means that the dimension of the column vector it will be equal to ${\left(1\times 20\right)}^{T}$.

**Figure 3:**
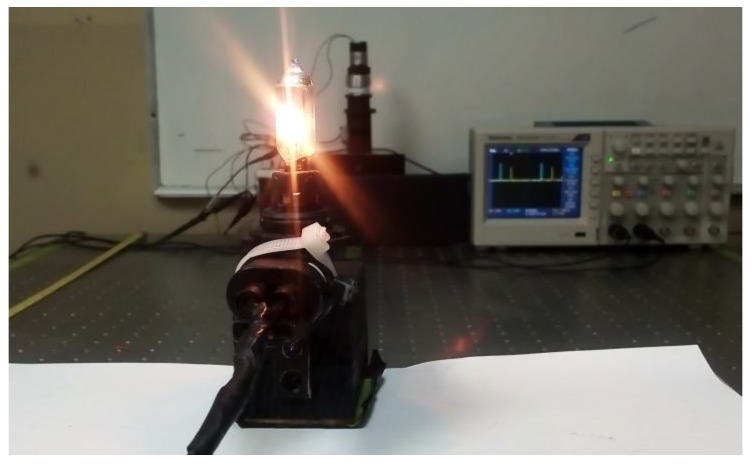
This figure shows a light bulb that is used as a source of radiation and the output of the Optical Scanning System (OSS) displayed by an oscilloscope.

The photosensors, such as photodiode, phototransistor, LDR, and Infrared LED was capable to sense the light bulb that is illustrated in Figure 3.

**Figure 4:**
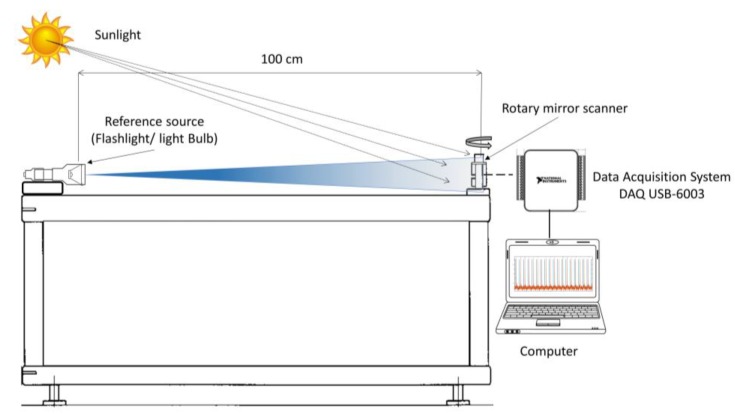
This figure shows a flashlight used a source of radiation and the output of the OSS displayed acquired by data acquisition (DAQ) and displayed at the computer.

The experimental set-up for the optoelectronic sensor evaluation that was used in this paper is presented in Figure 4. The main elements are used with environmental conditions are: a reference source, OSS, data acquisition (DAQ’s system), and a computer to process the signal that was captured. This figure illustrates the OSS system at 90° with respect to the source of light, in this example, a flashlight is used. However, it used a blue lamp as energy source and a LED as a photosensor with a maximum response at 420 nm wavelength. The reason for choosing a light bulb as energy source for the testing process is due to its wide spectral range to radiate energy to the phototransistor, LED, and photodiode that were used in this work. For example, the best response of the infrared LED and phototransistor is around 940 nm and the spectral response of the used LDR is around 600 nm wavelength.

## 4. Results

This section describes the experiments that were done in the laboratory and the real operational environment. The data in Tables 2, 4, 6, and 8 are from experiments that are realized only in laboratory, where the samples were taken from the OSS, as following: first, each sample had a duration of 10 s. The parameters and type of input of the DAQ system were sampled using a 50 KHz sampling frequency and two analog input channels were used for data acquisition. The motor frequency of the OSS was 20 Hz. In order to realize the experiment, three configurations were taken, such as changing the position of the reference source.

The first configuration of the experiment was called Position 90°, and the second and third configuration of the experiment were 91° and 92°, respectively. Position 90° means that blue flashlight was situated at 90° with respect to the position of the OSS at a distance of 1 m. Each sample was taken during 10 s in duration, for example in sample 1, the standard deviation σ_1_ and mean µ_1_ were calculated. Also, it should be emphasized that each sample corresponds to the angles calculated by the OSS in a controlled environment. All data in the Tables 2–5 were calculated by developing an algorithm in MatLab and comparing the real angle of the source (90°, 91°, and 92°) with respect of the angle that was detected by the algorithm of SA by returning one column vector each number of sample. The total amount of time for all of the configurations was 300 s, with 100 s corresponding to each configuration. The column vector, called Sample1, has $m_{DAQ}=217$ elements, if we multiply the number of pulses of the motor (20 pulses/seconds) by the time that was sampled by DAQ (10 s), we note that the number of angles calculated is $m_{theorical}=220$, which is not equal to the 217 elements calculated. This is due to the motor frequency was not controlled. Each sample is saved in Equation (6), as following.

(6) $$Sample1={\left[\begin{array}{ccc} 90.537 & 90.494 & 90.581 \end{array}\ldots \begin{array}{ccc} Sample1_{\left(m-2\right)} & Sample1_{\left(m-1\right)} & Sample1_{\left(m\right)} \end{array}\right]}^{T}$$

Table 2 shows the experiment with blue LED in a controlled environment. For this experiment, the results with blue LED as a sensor can be appreciated, each sample was taken during 10 s and the motor frequency of the OSS was 20 Hz. In position 90°, the minimum standard deviation that was obtained from the total samples was σ = 0.1°, which was calculated from a row vector with 200 angles that were captured by the OSS during 10 s sample time. For the position 91° and 92°, the minimum standard deviation obtained was 0.08 and 0.13, respectively.

**Figure 5:**
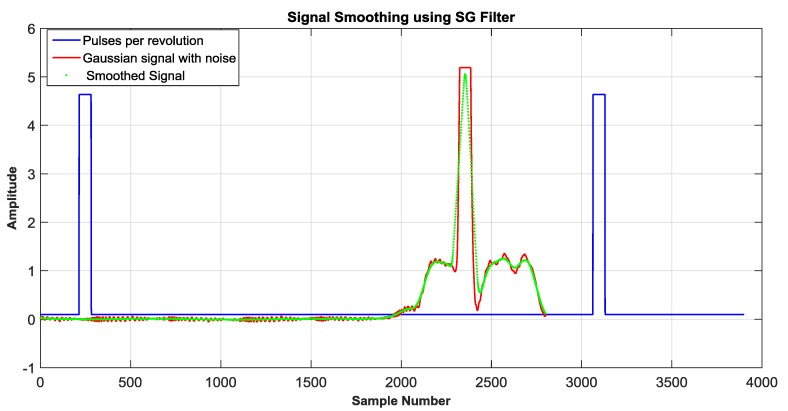
Signal smoothed (green) by using digital filter by using a blue LED as a photosensor.

On the other hand, the blue LED was confronted to the adverse conditions caused by the sunlight. The signal captured from the blue LED was smoothed using a Savitzky-Golay filter (SG) [18]. Recalling the purpose of this paper, which is defined by the comparison between different photosensors In Figure 5 the signal generated by the blue LED is illustrated, note that signal in red color is the output of the OSS system without a filter. The signal in green color represents the signal filtered using the SG filter.

**Figure 6:**
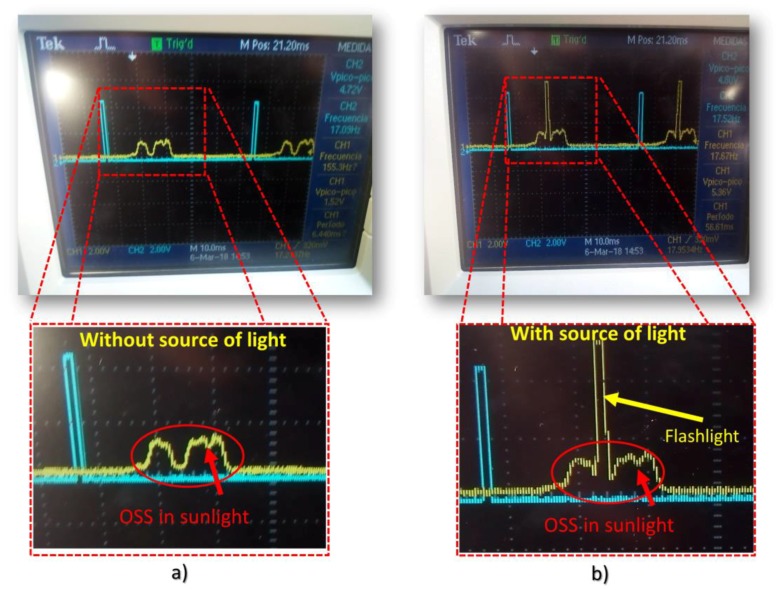
The output signal from a blue LED as a photosensor are displayed by oscilloscope. (**a**) These figures illustrate the output from a LED as a photosensor exposed to environmental conditions without source of radiation; (**b**) These figures show the output from the LED used as photosensor by using a flashlight as a source of radiation in sunlight.

In Figure 6a, the signal that was displayed by the oscilloscope is presented. Note that, in this case, the flashlight was turned off in order to compare when this is turned on. The voltage peak without flashlight was 1.52 V, while using a flashlight it was turned on the OSS could measure a voltage peak of 5.36 V, see Figure 6b.

Table 6 summarizes statistical data from the experiment using a blue LED as a photosensor and a blue LED flashlight as an energy source. The statistical data were calculated, as follows. First, it was taken a sample with duration of 10 s for each position of the reference source, and then the number of peak sample of the Gaussian signal generated by the blue LED was detected. These numbers of samples were recorded using a column vector. By knowing the number of samples of the rising and falling edge that was provided from the pulses that were generated by the opto-interrupter placed in the motor, these samples are recorded in another column vector. With these column vectors, the angles are calculated, due to the period are known by the numbers of samples when the pulses are measured. Since the full period represents a cycle of 360°, the next Equation (7), can be used:

(7) $$\alpha =\frac{Gaussian\_peak\_(\#.sample)}{Signal\_period(\#.sample)}\times 360$$

It must be noted that the motor frequency was not controlled, for this reason in Table 3 at 90°, 91°, and 92°, the number of samples to calculate the frequency of motor is decreased. According to the data from this table, the difference is less than 0.06°, when considering the average calculated for the angles selected for the experiment such as 90° and 91°, which the results were 89.94°, 91.97° versus the correct angles 90° and 92°. However, the difference between the correct angles 91° and the virtual angle calculated 91.53°, resulting in 0.53°. It is clear that the variance decreases considerably when the signal is filtered in order to smooth the data. For example, for the position 90°, the variance calculated was 8054.03 without filter and 0.02 using a filter.

In Table 4, the results with Infrared LED as a sensor used in laboratory conditions can be appreciated, each sample using a 10 s duration and the motor frequency of the OSS was 20 Hz. Notice that in this experiment, the resolution of the system has decreased in comparison to the experiment with blue LED due to 2.4 V peak of the infrared LED versus 4.8 V peak of the blue LED. In position 90°, the minimum standard deviation that was obtained from the total samples was σ = 0.17°, which was calculated from a row vector with 200 angles that were captured by OSS with a duration of 10 s each sample. For position 91° and 92°, the minimum standard deviation that was obtained was 0.14° and 0.17°, respectively.

**Figure 7:**
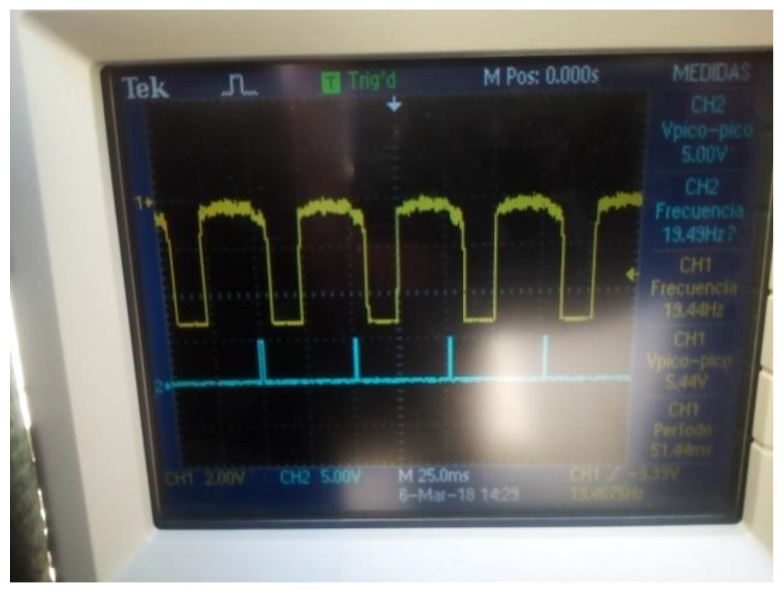
Signal captured using an infrared LED as photosensor of the OSS in the real environment.

In the following Figure 7, it can be appreciated that the infrared LED was saturated by sunlight, thereby this experiment could not be realized. However, in laboratory conditions, the infrared LED works without problems.

**Figure 8:**
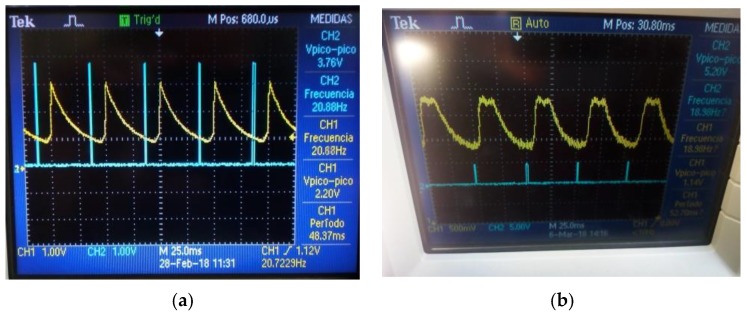
Signal captured by using a LDR as photosensor of the OSS, in (**a**), the output was taken from a controlled environment such as laboratory. In the (**b**), is illustrated the output that was generated in adverse conditions.

Note in Table 5, that the results in term of average and standard deviation were taken using a LDR as a photosensor with sampling time $T_{S}=10\;s$ and the motor frequency of $f_{M}=20\;\mathrm{Hz}$. The resolution of the OSS is about 1°, according to the results that are shown in this table. Figure 8 shows the output from the LDR it can be appreciated, that the rising and falling time of this sensor is different. However, the results that were obtained with this photosensor can be considered in order to develop an application, where the response time is dispensable. In the position 90° the minimum standard deviation obtained was σ = 0.14° calculated from a row vector with 200 angles captured by OSS during 10 s each sample. For the position 91° and 92°, the minimum standard deviation obtained was 0.20° and 0.26°, respectively.

The next Figure 8a shows the experimentation that was carried out in a controlled environment, such as laboratory, and the Figure 8b illustrates the output that was generated by LDR with the interference of the sunlight. Note that LDR cannot sense the reference source due to the exposure to the sunlight.

Table 6 shows the results of the experiments using a phototransistor as photosensor and a bulb light as reference source, operating in a controlled environment. The results in terms of standard deviation from position 90°, 91°, and 92° are 0.2, 0.14, and 0.32, respectively. On the order hand, the best results for the mean value are 90°, 91.13°, and 92.12°, respectively.

**Figure 9:**
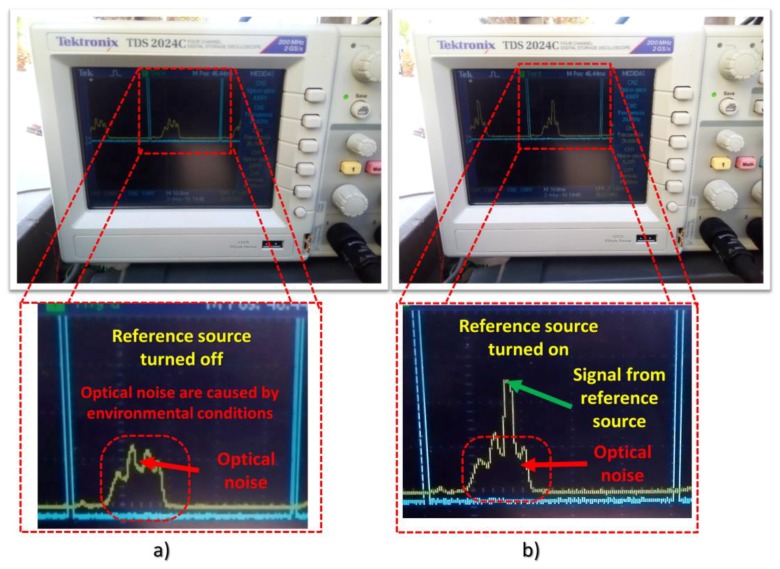
Signal captured by using a phototransistor. (**a**) The phototransistor detects peaks of voltage caused by environmental factors (external agents such as temperature and other source of radiation); (**b**) In spite of the sun's radiant energy, the system OSS could detect the bulb light.

Once the parameters of standard deviation and mean are established from the experiment that was realized in the laboratory, the next step was to experiment in a place exposure to environmental agents, such temperature and dust, especially with sunlight. Figure 9 illustrates the experiment that was realized in a real operation environment using a phototransistor as photosensors. According to Figure 9a, the OSS is detecting the reflection of the sunlight on the surfaces from the environment. For this case, the bulb light was turned off in order to make it possible to distinguish between the different external sources of light that were caused by the sunlight. Figure 9b shows the signal that was detected by the OSS when the bulb light was turned on.

**Figure 10:**
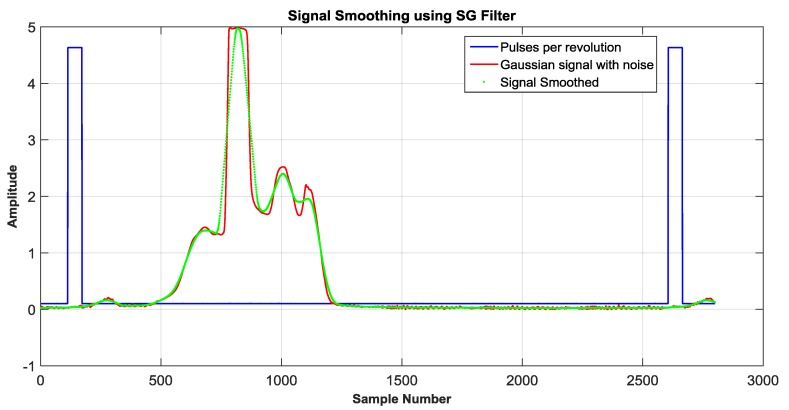
Signal captured using a phototransistor as photosensor in the OSS.

After the signal was detected by inspection, as mentioned before, the signal was acquired using a DAQ with 50 kHz sampling rate and a DC motor with angular frequency of 20 Hz. The reference source was placed at 90°, 91°, and 92° with respect to the OSS. Figure 10 reveals that the Gaussian signal that was generated by the phototransistor was clipped off at 5 V, due to the gain of this photosensor exceeds the power supply.

Table 7 shows the statistical data that were calculated from phototransistor and provides an estimation of when the angles between the OSS and light of source have been calculated. There are two columns for each angle, where the first column contains data processed without a filter, and the second column contains data smoothed using the Savitzky-Golay filter. The difference is considerable when the signal is filtered. For example, at the angle 90°, the system calculated an angle of 189.13°. However, the real angle is 90°, resulting a difference of −99.13°, on the other hand, the difference is reduced to 0.39° when the signal is smoothed by the digital filter.

**Figure 11:**
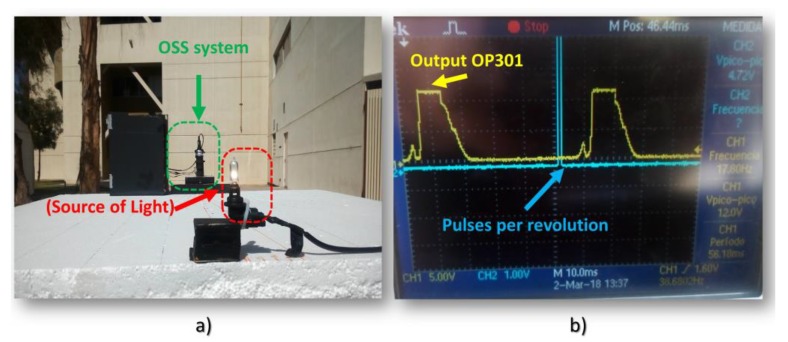
Data acquisition by using OPT301 in a real operation: (**a**) This figure shows the OSS system and bulb light as energy source and OPT 301 as a photosensor in the real operational environment; and, (**b**) Oscilloscope display the signal generated by pulses of ITR8102 (turquoise) and Gaussian signal (yellow) from sunlight.

As it can be seen from Figure 11, it was not possible to detect the signal in the real operational environment generated reference source (bulb light) by using the photodiode OPT301 as a photosensor, due to the interference with sunlight. Figure 11 illustrates the experiment that was realized in sunlight and the output from OPT301 that was displayed by the oscilloscope. Figure 11a shows the OSS, the source of light (light bulb), and Figure 11b illustrates the signal that was displayed by the oscilloscope. It must be noted that the output in yellow color is caused by reflections of the sunlight. The results from the photodiode OPT301 in laboratory are shown in Table 8.

## 5. Conclusions

The comparison between different types of photosensors has been presented in order to select a photosensor, which can be employed in a hostile environment where naturals and external light sources are presented. The photosensors that can detect the reference light source exposed to sunlight conditions, are visualized in the Table 9. The results with the phototransistor and LED show that it is possible to discriminate the reflection of the sunlight with the support of digital filters, such as FIR filters. Under sunlight conditions, using the blue LED gives satisfactory results, including a standard deviation of 0.16 and an average of 89.94 at 90° versus a standard deviation of 0.138 and an average of 89.83° for the same angle of reference. The results from the blue LED as a sensor and phototransistor that was obtained from both the laboratory and outdoors can be considered to be applied by OSS with adverse conditions. It is important to mention that the error in angle measurements for the blue LED in sunlight conditions were better (0.06°) than the experiments that were carried out under laboratory conditions (0.17°). The reason for this difference is due to the signal sensed in the adverse environment had to be smoothed by digital filter.

All of the photosensors were placed at a distance of 1 m with respect to the light source. However, in the case of the gain of the phototransistor at this distance, the output was clipped off because the voltage gain of this photosensor exceeded the power supply. According to the datasheet, the peak response wavelength is at 940 nm, while considering that the literature part of this region is blocked by atmospheric water vapor that is centered at this wavelength. Furthermore, the phototransistor contains a daylight blocking filter in order to reduce the sunlight radiation. The use of the blue LED in environmental conditions resulted that this device can attenuate the radiation that is generated by the sunlight at 2 V, and can detect our reference of source that was used in the experiments. It is important to mention that gain of the blue LED and phototransistor was clipped off at 5.2 V because of the power supply limitation. It is clear, that it is possible to avoid the saturation of the op amp by incrementing the distance between the OSS and the source of light. However, it was not done for reasons of standardization of the experiments.

On the other hand, the devices, such as OPT301, LDR, and infrared LED, cannot detect the reference of source, because the sunlight saturated the sensor according to their spectral responsivity, see Table 9. The best response of the photodiode OPT301 is the over spectral range (700 nm to 800 nm) according to a datasheet in the infrared region. In the region of visible light, the OPT301 has high responsivity at 650 nm 0.47 A/W. However, it cannot detect the light bulb because of interference with sunlight. This device can be limited to work in real-life environmental conditions, but we can get satisfactory results in a controlled environment, such as a laboratory. When the LDR was used and infrared for the experiment out of the laboratory the results were null.

The perspective to work is controlling the angular velocity of motor due to the OSS accuracy and resolution depends on the stability of the measurement speed. Additionally, one of the main goals is to implement and develop a system that is based on microcontroller by employing digital filters that can be used for various practical applications, such as large engineering structures SHM, mobile robot navigation, remote sensing, etc.

## Figures and Tables

**Table 1:** Spectral parameters of different types of semiconductors.

Material	BandGap (eV)	Energy (J)	Wavelength, λ (nm)	Color
Germanium	0.66	1.056 × 10^−19^	1882	Near Infrared
Silicon	1.12	1.792 × 10^−19^	1109	Near Infrared
GaAs	1.43	2.288 × 10^−19^	868	Near Infrared
GaAsP	1.9	2.288 × 10^−19^	654	Visible (Orange)
CdSe	1.70	2.723 × 10^−19^	729	Visible (Red)
CdS	2.42	3.877 × 10^−19^	512	Visible (Green)
ZnSe	2.7	4.320 × 10^−19^	460	Visible (Blue)
GaN	3.4	5.440 × 10^−19^	365	Visible (Violet)
InSb	0.17	0.2723 × 10^−19^	7.29	FIR
PbS	0.41	0.656 × 10^−19^	3.026	NIR

Source: Sze, S.M., Physics of Semiconductor Device, Wiley Interscience Publication, 1981, pp. 848–849.

**Table 2:** Experiment in a laboratory using one blue LED as light source and one LED used as photosensor.

# Samples	Position 90°		Position 91°		Position 92°	
	σ_1_	µ_1_	σ_2_	µ_2_	σ_3_	µ_3_
1	0.13	89.54	0.12	90.95	0.28	92.01
2	0.12	89.76	0.12	90.86	0.25	92.10
3	0.16	89.72	0.15	91.03	0.23	92.07
4	0.13	89.97	0.28	90.56	0.19	92.19
5	0.15	89.79	0.19	90.53	0.23	92.40
6	0.17	90.11	0.15	90.78	0.13	92.44
7	0.13	90.16	0.17	90.61	0.17	91.90
8	0.14	89.93	0.17	90.59	0.29	91.60
9	0.15	89.76	0.13	90.83	0.13	91.69
10	0.10	89.56	0.08	91.04	0.13	91.69

**Table 3:** Statistical data from blue LED used as photosensor in a real environment.

# Statistical Data	Position 90°		Position 91°		Position 92°	
	Without Filter	Smoothed by SG	Without Filter	Smoothed by SG	Without Filter	Smoothed by SG
Average	186.31	89.94	185.62	91.53	188.55	91.97
Standard deviation	89.74	0.16	86.08	0.11	87.13	0.11
Variance	8054.03	0.02	7410.63	0.013	7593.19	0.013
Samples	1135	174	1133	172	1068	171
Min. Value	0.35	89.59	0.35	91.04	0.35	92.59
Max. Value	359.12	90.34	359.14	91.88	359.16	93.25

**Table 4:** Experiment in a laboratory with bulb source light and LED infrared used as photosensor.

# Samples	Position 90°		Position 91°		Position 92°	
	σ_1_	µ_1_	σ_2_	µ_2_	σ_3_	µ_3_
1	0.43	90.56	0.35	91.02	0.22	91.27
2	0.28	90.55	0.33	90.92	0.30	91.54
3	0.44	90.26	0.31	90.76	0.18	91.06
4	0.28	90.46	0.25	91.09	0.34	91.55
5	0.25	90.40	0.48	90.44	0.35	91.70
6	0.19	90.38	0.34	90.35	0.28	91.51
7	0.38	90.31	0.39	90.16	0.17	91.12
8	0.40	90.43	0.14	90.46	0.28	91.39
9	0.17	90.33	0.20	90.41	0.43	91.41
10	0.23	90.32	0.28	90.62	0.30	91.44

**Table 5:** Experiment in a laboratory with bulb source light and LDR used as photosensor.

# Samples	Position 90°		Position 91°		Position 92°	
	σ_1_	µ_1_	σ_2_	µ_2_	σ_3_	µ_3_
1	0.14	89.61	0.26	90.62	0.26	91.74
2	0.35	89.90	0.20	90.76	0.34	91.48
3	0.24	89.66	0.55	91.28	0.25	91.56
4	0.35	89.66	0.30	90.64	0.25	91.56
5	0.46	90.02	0.42	90.63	0.26	91.61
6	0.16	89.95	0.41	90.77	0.26	91.55
7	0.19	89.91	0.33	90.89	0.35	91.66
8	0.25	89.43	0.43	90.88	0.51	91.90
9	0.17	89.78	0.33	90.73	0.30	91.81
10	0.33	89.71	0.29	90.80	0.35	91.59

**Table 6:** Experiment in a laboratory with bulb source light and Phototransistor used as photosensor.

# Samples	Position 90°		Position 91°		Position 92°	
	σ_1_	µ_1_	σ_2_	µ_2_	σ_3_	µ_3_
1	0.30	90.18	0.34	91.16	0.48	92.44
2	0.28	90.00	0.23	91.33	0.32	92.12
3	0.20	90.25	0.27	91.30	0.33	92.33
4	0.20	90.33	0.14	91.33	0.33	92.41
5	0.29	90.35	0.28	91.13	0.43	92.18
6	0.29	90.10	0.34	91.22	0.29	92.61
7	0.41	89.62	0.44	90.90	0.51	92.66
8	0.34	89.80	0.42	91.13	0.59	92.67
9	0.46	89.93	0.56	91.36	0.43	92.58
10	0.48	90.23	0.32	91.36	0.37	92.41

**Table 7:** Statistical data from phototransistor used as photosensor in a real environment.

# Statistical Data	Position 90°		Position 91°		Position 92°	
	Without Filter	Smoothed by SG	Without Filter	Smoothed by SG	Without Filter	Smoothed by SG
Average	189.13	89.61	189.81	90.72	193.72	91.78
Standar deviation	100.26	0.15	101.49	0.22	105.20	0.19
Variance	10052.19	0.02	10301.53	0.048	11068.63	0.039
Samples	1081	200	971	200	823	196
Min. Value	0.39	89.19	0.40	90.32	0.38	91.54
Max. Value	358.97	90.39	358.96	91.49	359.01	92.36

**Table 8:** Experiment in a laboratory with bulb source light and photodiode OP301 used as photosensor.

# Samples	Position 90°		Position 91°		Position 92°	
	σ_1_	µ_1_	σ_2_	µ_2_	σ_3_	µ_3_
1	0.23	90.40	0.18	91.81	0.20	92.65
2	0.21	90.46	0.14	91.57	0.21	92.92
3	0.18	90.46	0.19	91.68	0.19	92.80
4	0.29	90.34	0.23	91.49	0.29	92.11
5	0.17	90.29	0.27	91.65	0.14	92.84
6	0.26	90.27	0.23	91.63	0.13	92.81
7	0.20	90.44	0.28	91.89	0.14	92.56
8	0.20	90.45	0.20	91.52	0.12	92.73
9	0.16	90.37	0.12	91.34	0.16	92.78
10	0.25	90.49	0.12	91.42	0.11	92.62

**Table 9:** Experiment in a laboratory with bulb source light and photodiode OP301 used as photosensor.

**Laboratory Test**						
	**Position 90°**		**Position 91°**		**Position 92°**	
	**σ**	**µ**	**σ**	**µ**	**σ**	**µ**
OPT301	0.215	90.397	0.196	91.6	0.169	92.682
Blue LED	0.138	89.83	0.156	90.778	0.206	92.009
Infrared LED	0.305	90.4	0.307	90.623	0.285	91.399
LDR	0.264	89.763	0.352	90.8	0.313	91.646
Phototransistor	0.325	90.079	0.334	91.222	0.406	92.441
**Sunlight Conditions**						
	**Position 90°**		**Position 91°**		**Position 92°**	
	**σ**	**µ**	**σ**	**µ**	**σ**	**µ**
OPT301	*		*		*	
Blue LED	0.16	89.94	0.11	91.53	0.11	91.97
Infrared LED	*		*		*	
LDR	*		*		*	
Phototransistor	0.15	89.61	0.22	90.72	0.19	91.78

* The OSS not detected the signal caused by source of reference.
